# A Qualitative Assessment of Gender- and Race-Related Stress Among Black Women

**DOI:** 10.1089/whr.2021.0041

**Published:** 2022-02-14

**Authors:** Meghan Tipre, Tiffany L. Carson

**Affiliations:** ^1^ Division of Preventive Medicine, School of Medicine, University of Alabama at Birmingham, Birmingham, Alabama, USA.; ^2^ Division of Population Sciences, Department of Health Outcomes and Behavior, H. Lee Moffitt Cancer Center and Research Institute.

**Keywords:** Black women, psychological chronic stress, obesity

## Abstract

***Background:*** Chronic psychological stress has been associated with several adverse health outcomes, including obesity. Black women report higher levels of psychological stress than White women and carry a disproportionate burden of chronic conditions associated with psychological stress, including obesity. Research also suggests that in addition to generic stressors, Black women also experience race- and gender-related stress. To further explore this, we conducted structured focus groups to examine Black women's perspectives about stress.

***Materials and Methods:*** Using the nominal group technique, three sessions (total, *n* = 33) were conducted exclusively with Black women to solicit responses to the following questions: (1) What are the top sources of stress for women? (2) What are the top stressors specifically for Black women? and (3) How do these stressors affect weight? Using a systematic method, participants ranked responses in order of importance. Responses were compiled and tabulated to identify which statements were viewed as most important by respondents.

***Results:*** Mean age and body mass index of participants were 43.0 ± 10.1 years and 35.1 ± 7.9 kg/m^2^, respectively. The top 3 responses for question 1 were health, family, and relationships. Safety of children and raising Black children, being the head of the family, and finances were the top responses for question 2. Emotional eating, not enough time for exercise, and eating fast food due to lack of time or money were voted as the top reasons as to how stressors influence women's weight.

***Discussion:*** Our findings are consistent with previous work reporting that, along with generic stress, race- and gender-related stress contribute to the overall experiences of Black women. This work adds qualitative depth to allow for a better understanding of the unique sources of stress for Black women. These gender- and race-related stressors should be considered when offering stress management interventions for weight loss and general health promotion among Black women.

## Introduction

In the United States, perceived chronic stress disproportionately affects Black women exacerbating poor health outcomes. According to Tsigos et al., stress is defined as a state of disharmony, that is, *cacostasis or allostasis* and is counteracted by a range of physiologic and behavioral responses, which aim to maintain/reestablish the threatened homeostasis (adaptive stress response).^1^ Under normal conditions, these responses are adaptive and improve the chances of survival. However, high-intensity and/or chronic stress can reduce body's ability to adapt and thus lead to disease. Stress response is typically mediated through a complex system, including the neuroendocrine, molecular, and cellular infrastructure and is determined by a multitude of individual factors, including genetic, environmental, and developmental factors.

Because response to stress involves several coping mechanisms, dysregulation, that is, hypo- or hyperactivation as a result of potent and/or chronic stress, can have detrimental effects on physiological systems, including central nervous system, cognition and learning, immune system functions, cardiovascular systems, gastrointestinal complications, and endocrine system.^2^ Evidence from animal and human studies have linked allostatic load, a biological measure for stress, with numerous negative health outcomes, including decreased cognitive and physical function, heart disease, stroke, diabetes, and mortality.^3^

Studies have observed that Black women may be excessively burdened by physiological impacts of chronic stress caused by health disparities associated with chronic stressors, including perceived discrimination, neighborhood stress, daily stress, family stress, acculturative stress, environmental stress, and maternal stress.^4^ They are likely to suffer the twofold consequences of social stress resulting from the interaction between racial and gender discrimination compounded by health and socioeconomic disparity. This may ultimately contribute to an increase in disease manifestation.

National statistics indicate that Black Americans have worse health outcomes than non-Hispanic White Americans. Black Americans are 77% more likely to be diagnosed with diabetes than their white peers. Similarly, rates of cardiovascular diseases, obesity, high blood pressure, and cancers are much higher among Black individuals compared with their White peers.^5^ Yet, there is limited research among Black women to find out about their sources, perceptions, or beliefs regarding chronic stress. Understanding the sources of stress and its role in disease pathology is key to developing tailored treatment plans for individuals that include both pharmacological (medications and/or nutraceuticals) and nonpharmacological (change in lifestyle, daily exercise, healthy food choices, *etc.*) interventions.

Thus, we conducted a pilot qualitative study to assess Black women's perspectives about stress and to explore the intersection of race and gender in the stress experience of Black women.

## Materials and Methods

A convenience sample of Black women over the age of 18 was recruited to participate in highly structured focus groups that employed the Nominal Group Technique (NGT). Recruitment approaches included dissemination of flyers containing study details *via* e-mail to staff members at the University of Alabama at Birmingham and to participants of previous research studies of this study's Principal Investigator in the Birmingham Metropolitan area, and word of mouth. Potential participants were screened by telephone. Interested individuals were asked to verify their race, ethnicity, and age.

Eligibility criteria included women who identified themselves as Black or African American, were 18 years or older, and willing to participate in the study. Women who did not understand English language were excluded. The ethics approval for the pilot study was obtained from the Institutional Review Board at UAB. Three sessions were conducted over a period of 2 weeks. Each session was for 1 hour and a $25 gift card was given as incentive.

**FIG. 1.**
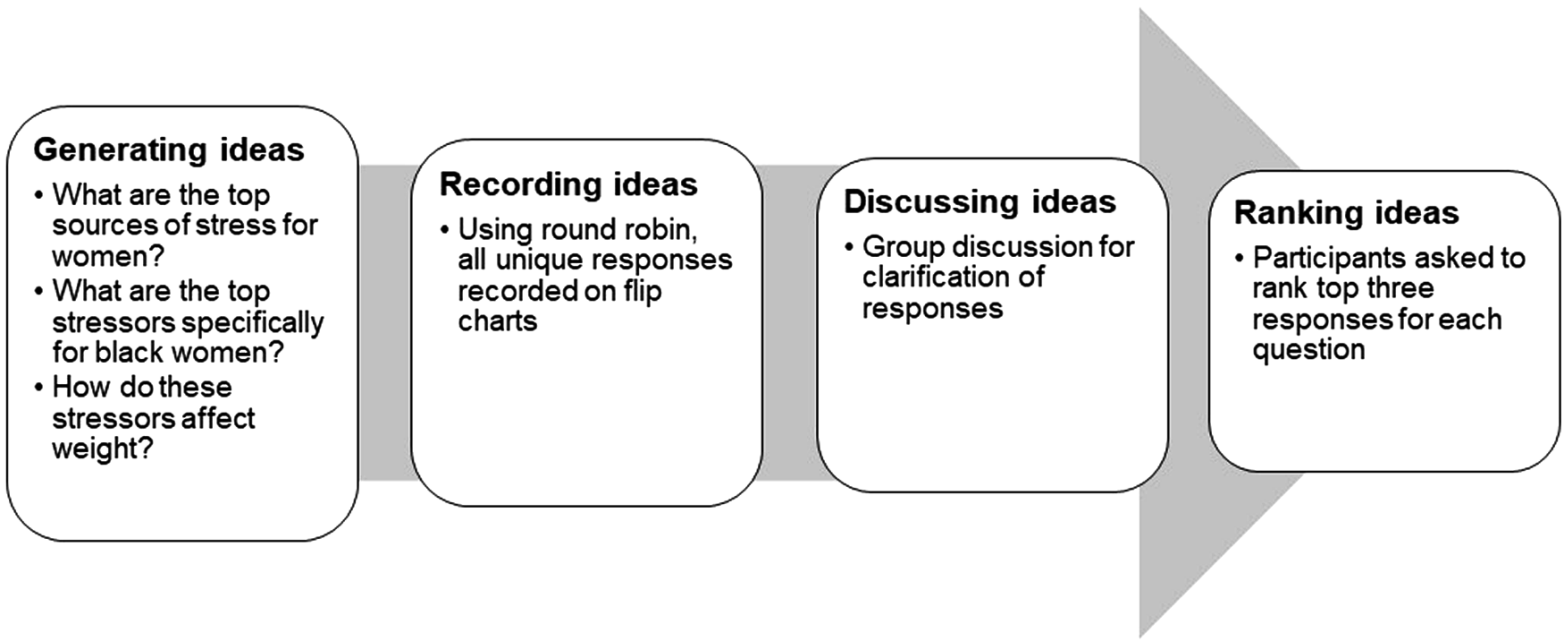
Steps in Nominal Group Technique.

The NGT was used to conduct the focus groups. NGT is a modified focus group method designed by Delbecq and Van de Ven,^6^ and is typically used to generate potential answers to questions agreed upon *a priori.* The method consists of four major steps, including (Fig. 1): (1) idea generation—the moderator of the focus group poses questions or the problem to the participants. Each member is given a set amount of time to think of ideas in response to the posed question and write down as many ideas as possible; (2) round robin—in round robin fashion, the participants are asked to express their ideas one at a time. The moderator records the ideas on a flipchart. No discussion occurs until this point. In case a participant has an idea that is already expressed by a previous participant, then the participant is asked to skip that idea and express the next response he or she has written down.

The participants continue to express their ideas until everyone has listed their ideas on the flipchart. (3) Discussion and clarification of ideas—the moderator facilitates the discussion and everyone is asked to review all ideas listed on the flipchart to ensure that they understand the ideas. The participants are encouraged to ask questions and clarification of any ideas that are not clear. At this point, the originator may provide an explanation and if needed, can change the wording of the idea. (4) Prioritize and rank the ideas—Once the ideas are finalized, the participants are asked to write down ideas that they perceived as priorities considering the original question. Furthermore, they are asked to rank the ideas in order of their significance. The participants are given a set time to prioritize and rank the ideas. This is the last step of NGT.

In our study, ideas that received the highest number of votes were considered as most salient. Sessions lasted ∼60 minutes. Upon arrival, the session moderator read a script detailing the purpose for the meeting and describing the process to expect. Participants were told that they were being asked to provide their perspectives on three questions related to stress and eating specifically as it relates to women. Before starting the NGT session, we administered a brief demographic survey consisting of five questions to elicit information on age, marital status, income, education level, and employment followed by the Perceived Stress Scale (PSS)-10 to assess short-term perceived chronic stress among the participants. This instrument measures the degree to which an individual perceives life events in the previous month to be stressful.

Sample items include: “In the past month, how often have you felt that you could not cope with all the things that you had to do?” and “How often have you felt that you were unable to control the important things in your life?” Responses are on a 5-point Likert sale (0 = never to 4 = very often). Four items (*e.g.*, “felt confident about ability to handle personal problems”; “I felt things were going my way”) are positively worded and were reverse coded for analysis. Higher total scores correspond to greater perceived stress. The PSS-10 has been validated in diverse populations and demonstrated to have good internal consistency (α = 0.77) in a previous study conducted by our group among a similar population.^7^

During the NGT sessions, we solicited responses for three questions: “1. What are the top sources of stress for women?”; “What are the top stressors specifically for Black women?” and “How do these stressors affect weight?” Participants were given 5 minutes for idea generation for each of the three questions. A research assistant listed all the responses from the participants elicited using the round robin method. After clarification of the ideas, each participant was asked to rank the top 3 ideas that they considered important in response to each of the three questions on flashcard. All data were collected and entered in excel. Data were imported in SAS for further analyses.

Descriptive statistics were computed to describe the characteristics of the participants. Ranked responses were analyzed to evaluate which ideas received top priority by the participants across all the three groups. All unique responses are presented in Supplementary Table ST1. In addition to the surveys and the NGT, we obtained height (in cm) and measured participant's weight (in pounds) using an electronic weighing machine to estimate body mass index (BMI).

## Results

Table 1 describes the sociodemographic characteristics of the participants. Overall, we conducted three focus group sessions with a total of 33 participants; 10 participants in sessions 1 and 2, and 13 participants in session 3. All participants were Black women with an average age of 43 ± 10 years. Almost 73% of the women had a BMI of greater than or equal to 30 kg/m^2^. Roughly a third of women were either married (*n*, 13; 39%) or single (*n*, 11; 33%), and had a graduate (*n*, 10; 30%) or a Bachelor's degree (*n*, 9; 27%). About 66% had income $60,000 or more. Almost 58% (*n*, 19) had a perceived stress score of 13 or more, which indicates perceived stress at or above the national median when the survey was originally validated.^7^ More recent validation studies have reported the mean PSS-10 score to be 16 among women and Black individuals.^8^

**Table 1.** Demographics (*N* = 33)

	*N* (%)
Total participants	33
Age (mean, SD) (in years)	43 (10)
BMI (mean, SD) (kg/m^2^)	35 (7.9)
BMI categories	
18.5–24.9	2 (6.1)
25–29.9	7 (21)
≥30	24 (73)
Marital status	
Living with partner, but not married	1 (3.0)
Married	13 (39)
No longer married (divorced, widowed)	6 (18)
Separated	1 (3)
Single (never married)	11 (33)
Missing	1 (3.0)
Education	
High school/General education development	1 (3.0)
Associate degree	5 (15)
Some college	7 (21)
Bachelor's degree	9 (27)
Graduate or professional degree	10 (30)
Missing	1 (0.3)
Household members	
1	4 (12)
2	11 (33)
3	11 (33)
4	2 (6.1)
5	4 (12)
Income	
$20,000–$39,999	4 (12)
$40,000–59,999	4 (12)
$60,000–$79000	9 (27)
$80,000 or more	13 (39)
Less than $20,000	1 (3.0)
Missing	2 (6.1)
Perceived stress scale score	
Mean (SD)	17 (6.8)
Median	17
Perceived stress scale	
0–13	12 (36)
13–40	19 (58)
Missing	2 (6.1)

BMI, body mass index.

Participants provided 52 unique responses as top sources of stress for women in general in response to question 1, 71 responses as sources of stress related specifically to Black women in response to question 2, and 29 responses as to how stressors relate to weight in response to question 3.

The results rankings are provided in Table 2. In response to the question “What are the top sources of stress for women?” the top 3 responses were health, family, and relationships.

**Table 2.** Summary of the top 3 responses to questions presented in nominal group technique sessions

Questions	Top 3 ranked responses
What are the top sources of stress for women?	Health Family Relationships
What stressors relate specifically to Black women?	Safety of children/raising black kids Being head of household/single parenting Finances
In what ways do these stressors influence women's weight?	Emotional eating No time for exercise Eating fast food due to lack of time or money for healthy foods

For question 2, “What are the top stressors specifically for Black women?” the top 3 responses were safety of children and raising Black children, being the head of the family, and finances.

In response to the third question about how stressors affect weight, participants voted emotional eating, not enough time for exercise, and eating fast food due to lack of time or money as the top reasons as to how stressors influence women's weight.

## Discussion

In recent years, researchers have recognized the need to study the impact of stress among Black women as a function of their ethnic and gender interactions. While much of the narrative has been focused on the sources of stress among women in general, little has been studied about the unique experiences of Black women who face the intersectionality to identify with a gender and race that experience discrimination. The results of our qualitative study highlighted the differences between the sources of stress that are perceived as being related to women in general and those perceived as being unique to Black women.

Participants in our focus groups ranked health, family, and relationships as the most common sources of stress for women in general, whereas safety of children and raising Black children, being the head of the family, and finance, as the most common sources of stress among Black women. These findings are consistent with findings from the few studies that have investigated chronic stress and its impact on health among minority populations. Jones et al. evaluated the changes in perceived stress from late pre-menopause to postmenopause and identified significant life stressors perceived by a cohort of African American women.^9^

Results of the study revealed six categories of stress before and after menopause, including finances, caring for family members, relationships, personal health and aging, race and discrimination, and raising children. Jones et al. had a sample size of 15 women with an average age of 61 years and a mean PSS score of 17 (±6.4). In comparison, we had 33 women in our study with an average age of 43 ± 10 years and PSS score of 17 (6.8).

While the women in our study were younger, they had similar mean PSS scores, and the sources of life stressors identified in both the studies were similar. Studies among Black pregnant mothers have reported that emotional and psychological stress resulting from experiences of racial discrimination and financial worries were associated with negative birth outcomes.^10^ Other studies have reported combined effects of perceived social and physical disorders and perceived crime as major sources of stress associated with poor health outcomes, such as diabetes^11^ and cardiovascular diseases.^12^

The top stressors identified by our focus group participants are notable as they are consistent with phenomena like the Sojourner Syndrome^13^ or the Superwoman Schema^14^ that describe the multiple roles and social identities of Black women that are based in history that required Black women to be strong, self-reliant, and self-sacrificing in their roles as the caretaker of their families.^15,16^ Such roles are often times unwillingly taken on by them, possibly leading to complex adaptive patterns where needs of others take precedence over their own needs and requirements.^14–18^

Stressors can induce short-term responses, which may manifest as behavioral (*e.g.*, over eating),^19–21^ physiological (*e.g.*, elevated blood pressure or cortisol hormones),^22^ or psychological (*e.g.*, disturbed sleep).^23,24^ Repeated experiences of short-term effects could lead to irreversible long-term effects. Stress may be related to body weight and weight change through several mechanisms. People who overeat in response to stress are likely to choose foods that are high in fat and/or sugar content. High stress levels may also deter individuals from engaging in physical activity^23^ or result in sleep impairment, which may lead to enhanced appetite, cravings, and decreased motivation for physical activity.^20–23^

The racial disparity in the prevalence of obesity between White and Black Americans, particularly women could possibly be attributed to the unique and/or added stress Black women may experience in the context of U.S. society as compared with White women.^25^ When asked about how stressors could affect body weight, our study participants provided a wide range of options but voted emotional eating, not enough time for exercise, and eating fast food due to lack of time or money as the top reasons as to how stressors influence women's weight.

These observations suggest that the women in our focus group had increased awareness about the relationship between stress and unhealthy habits contributing to obesity. A baseline understanding and/or awareness about the impact of stress on health can be instrumental in designing behavioral interventions to manage stress.

Behavioral or psychosocial interventions that mediate the effect of stress may alter the short-term and long-term effects of chronic stress. These may include individual factors, such as coping behaviors, as well as social factors, such as access to material resources or social support.^11^ Similarly, interventions to reduce obesity among Black women cannot just focus on diet and physical activity alone but should also take into account management of stress. Findings from this study can be used to inform current behavioral weight loss interventions to include evidence-based stress management strategies as an approach to improve weight loss outcomes among Black women with elevated stress levels.

Our study had a few limitations, including a small sample size. Also, convenience sampling of participants and the qualitative design of the study limits external validity and generalizability of the study. Still, this approach allows for greater insight into perceptions that can be used to inform future studies and interventions.

## Conclusion

In conclusion, prior research has shown that Black women report higher psychological stress compared with other demographic groups in the United States. This study provides insight into how the intersectionality of two historically marginalized groups (*i.e.*, Black Americans and women) may result in unique stress experiences for Black women. These unique experiences should be considered when offering stress management strategies to improve the mental and physical health of this population.

## Supplementary Material

Supplemental data

## Supplementary Material

Supplementary Table ST1

## Abbreviations Used

BMI: body mass index
NGT: nominal group technique
PSS: perceived stress scale
