# Supplementary material for: A Qualitative Assessment of Gender- and Race-Related Stress Among Black Women
Source: Womens Health Rep (New Rochelle). 2022 Feb 14;3(1):222–7. doi: 10.1089/whr.2021.0041 (PMC8896166; doi:10.1089/whr.2021.0041)
Supplement: Supplemental data [file Suppl_TableST1.docx]

**Title: A qualitative assessment of gender- and race-related stress among Black women**

**Authors**

Meghan Tipre, DrPH, MSPH

Scientist I

Division of Preventive Medicine

School of Medicine

University of Alabama at Birmingham

Email: [mtipre@uabmc.edu](mailto:mtipre@uabmc.edu)

Tiffany L. Carson, PhD, MPH

Associate Professor

Division of Preventive Medicine

School of Medicine

University of Alabama at Birmingham

Email: [tiffanycarson@uabmc.edu](mailto:tiffanycarson@uabmc.edu)

**Corresponding author:**

Meghan Tipre, DrPH, MSPH

Scientist I

Department of Medicine

Division of Preventive Medicine

O’Neal Comprehensive Cancer Center at UAB

Office of Community Outreach and Engagement

UAB | The University of Alabama at Birmingham

MT 628 | 1717 11th Avenue South | Birmingham, AL 35294-4410

Phone: 205.934.6947

Fax:

Email: [mtipre@uabmc.edu](mailto:mtipre@uabmc.edu)

**Appendix I (Supplementary tables)**

**Table S1: Unique responses to study questions from participants**

|  | **Question 1: What are the top sources of stress for women?** |
| --- | --- |
|  | **Health** |
|  | **Family** |
|  | **Relationships** |
|  | Achievements |
|  | Body image |
|  | Weight |
|  | Work |
|  | Balancing work and family |
|  | Being a mother |
|  | Being a central resource for a lot of people |
|  | Finances |
|  | Children's safety and well being |
|  | Insecurities |
|  | Control |
|  | Healthy lifestyle, eating right |
|  | Caregiver (loved ones) |
|  | Lack of quiet time |
|  | Spouse |
|  | Lack of faith |
|  | Decisions |
|  | Lack of Self Care |
|  | Day to day hustle and bustle |
|  | Caregiver (parent role reversal) |
|  | Retirement - maintaining lifestyle |
|  | Single woman |
|  | Controlling calendar |
|  | Competition with other women |
|  | Church responsibilities |
|  | Household chores |
|  | Health and surgeries |
|  | Responsibility of teaching children about God |
|  | School - student loans |
|  | Child bearing years |
|  | Other people's problems |
|  | Single parent |
|  | Co-parenting |
|  | Society's view on beauty |
|  | Appearance |
|  | Life changes |
|  | Providing a safe place for children |
|  | Other people's expectations for marriage and children |
|  | Not coming out as angry/emotional |
|  | Excelling in your career |
|  | Not having acceptance from one of your parents |
|  | Pregnancy |
|  | Dealing with deaths |
|  | Opposite of what you taught your children |
|  | Feeling appreciated |
|  | Achieving personal and professional goals |
|  | Being desired by opposite sex (lack of being desired unrealistic) |
|  | Being treated respectfully |
|  | Social media |
| **Question 2: What are the top stressors specifically for Black women?** | |
|  | **Safety of children/Raising Black kids** |
|  | **Being single and a parent** |
|  | **Being head of household** |
|  | **Money/Income/Financial security** |
|  | Marriage |
|  | Know when to say 'No' |
|  | Perceptions about our behavior or demeanor |
|  | Career |
|  | Fear to meet our emotional and mental needs |
|  | Inherent inferiority |
|  | Caring for other families' children |
|  | Being considered unequal in a man's job |
|  | Appearance and weight |
|  | Best opportunities for kids |
|  | Colorism and standards of beauty |
|  | Disadvantages in health, disparity in access to healthcare |
|  | Decision maker in multiple situations |
|  | Recycled poverty - generational poverty |
|  |  |
|  | Complete caregiver |
|  | Hairstyles preferences and career implications |
|  | Image - body, societal, physical expectations |
|  | Self validation |
|  | Criticized for being too successful |
|  | Just being a Black woman |
|  | Lack of resources |
|  | Misunderstood |
|  | Racial slurs, comments |
|  | Being devalued by society |
|  | Jealousy among friends, coworkers |
|  | "Special" men - man with issues |
|  | Time management |
|  | Friendship |
|  | The Black man |
|  | Talking and listening |
|  | Dealing with white men |
|  | Work place biases and discrimination |
|  | Child births |
|  | Adult children |
|  | Balancing multiple stressors |
|  | Dealing with white women |
|  | Educating Black children due to lack of resources |
|  | Not enough rest |
|  | Balancing career and family |
|  | Boundary between parent and friend |
|  | Having to always be strong |
|  | Being grouped with and judged by other Black women |
|  | Fear of the unknown |
|  | Other's expectations and opinions |
|  | Bullying among children |
|  | Checking their children's rooms when they leave |
|  | Paying student loan |
|  | Taking care of elderly family members |
|  | Being an example for your children |
|  | Not being married and bear children by a certain age |
|  | Know the people you bring around your children |
|  | Getting promoted on the job |
|  | Income disparities vs. white women |
|  | Lack of mates due to Black men dating outside race |
|  | Raising fatherless children |
|  | High Black male incarceration |
|  | Being educated, articulate without being percceived being angry |
|  | Stereotyped by neighborhood |
|  | Being materialistic |
|  | Expected church duties |
|  | Not meeting your goals in timely manner |
|  | Being respected by our own people |
|  | Lack of family support |
|  | Black gay men (limit choices) |
|  | Systematic oppression |
|  | Depression |
|  | Age |
| **Question 3: How do these stressors affect weight?** | |
|  | **Not time for exercise** |
|  | **Emotional eating** |
|  | **Eating fast food regularly because of lack of time** |
|  | Elevate cortisol hormones |
|  | Inherited unhealthy eating |
|  | Late night eating and not sleeping |
|  | Chemical imbalance, eating times/frequency changed, more or less |
|  | Image related stress → camouflage our weight |
|  | Making dangerous decisions based on body image |
|  | Emotionally and physically drained - look old |
|  | Yo-yo dieting |
|  | Insomnia |
|  | Medically unnecessary procedures - enhancements, tummy tucks |
|  | Setting unrealistic weight goals for yourselves |
|  | Lack of interest |
|  | Health-medical reasons |
|  | Cause anxiety |
|  | lead to health issues |
|  | Cause depression |
|  | Drinking more alcohol |
|  | Unemployment |
|  | Working out too much |
|  | Low self-esteem |
|  | Stress |
|  | Cause people to try fad extreme diet |
|  | Weight - loss/gain |
|  | Decrease in activity |
|  | Missing meals |
|  | Overthinking |
